# Characterization of the Fishing Lines in Titiwai (=*Arachnocampa luminosa* Skuse, 1890) from New Zealand and Australia

**DOI:** 10.1371/journal.pone.0162687

**Published:** 2016-12-14

**Authors:** Janek von Byern, Victoria Dorrer, David J. Merritt, Peter Chandler, Ian Stringer, Martina Marchetti-Deschmann, Andrew McNaughton, Norbert Cyran, Karsten Thiel, Michael Noeske, Ingo Grunwald

**Affiliations:** 1 Ludwig Boltzmann Institute for Experimental and Clinical Traumatology, Vienna, Austria; 2 University of Vienna, Faculty of Life Science, Core Facility Cell Imaging & Ultrastructure Research, Vienna, Austria; 3 Technical University Wien, Institute of Chemical Technologies and Analytics, Vienna, Austria; 4 The University of Queensland, School of Biological Sciences, Brisbane, Queensland, Australia; 5 Spellbound Cave, Waitomo, New Zealand; 6 Department of Conservation, Wellington, New Zealand; 7 University of Otago, School of Medical Sciences, Department of Anatomy, Otago Centre for Confocal Microscopy, Otago, New Zealand; 8 Fraunhofer Institute for Manufacturing Technology and Advanced Materials, Department of Adhesive Bonding Technology and Surfaces, Bremen, Germany; Swedish University of Agricultural Sciences, SWEDEN

## Abstract

Animals use adhesive secretions in a plethora of ways, either for attachment, egg anchorage, mating or as either active or passive defence. The most interesting function, however, is the use of adhesive threads to capture prey, as the bonding must be performed within milliseconds and under unsuitable conditions (movement of prey, variable environmental conditions, unfavourable attack angle, etc.) to be nonetheless successful. In the following study a detailed characterization of the prey capture system of the world-renowned glowworm group *Arachnocampa* from the macroscopic to the ultrastructural level is performed. The data reveal that the adhesive droplets consist mostly of water and display hygroscopic properties at varying humidity levels. The droplet core of *Arachnocampa luminosa* includes a certain amount of the elements sodium, sulphur and potassium (beside carbon, oxygen and nitrogen), while a different element composition is found in the two related species *A*. *richardsae* and *A*. *tasmaniensis*. Evidence for lipids, carbohydrates and proteins was negative on the histochemical level, however X-ray photoelectron spectroscopy confirm the presence of peptides within the droplet content. Different to earlier assumptions, the present study indicates that rather than oxalic acid, urea or uric acid are present in the adhesive droplets, presumably originating from the gut. Comparing the capture system in *Arachnocampa* with those of orb-spiders, large differences appear not only regarding the silky threads, but also, in the composition, hygroscopic properties and size of the mucous droplets.

## Introduction

Adhesives are widespread in insects, used for different purposes (tarsal attachment, mating, nest building, egg anchorage, hunting, etc.) and secreted from different areas (abdomen, labium, tarsus) [1]. Because of this diversity, there are still many unexplored adhesive systems which differ significantly from the known and described systems and species:

Glowworms are the larval form of a fungus gnat from the family Keroplatidae [2], genus *Arachnocampa* [3], and are a popular tourist attraction due to their unique system of bioluminescence [4; 5; 6; 7; 8; 9; 10; 11], which has been extensively studied during the last 100 years [12; 13; 14]. The light organ at the larva’s posterior end is part of the Malphigian tubules system and the ventral and lateral surfaces are covered by a tracheal reflector to direct the light downwards [15]. The main function of this light system is to attract prey, captured subsequently by means of adhesive threads [7; 16]. The blue-green light is produced by a luciferin–luciferase system with ATP activated in the presence of Mg^2+^ [17]. The peak emission wavelength is 488 nm [17], producing a more bluish light than bioluminescent beetles (peak wavelength 560 nm) [18]. The *Arachnocampa* luciferase is a relatively small enzyme of 36 kDa [19] in relation to that of the related mycetophilid species, *Orfelia fultoni* (140 kDa) [19] or the well known firefly *Photinus pyralis* (62 kDa) [20].

In total, nine species are known within the genus *Arachnocampa*, all endemic to Australia and New Zealand [21; 22; 23; 24; 25]. While there is some morphological data available for the Australian species [24], the New Zealand endemic species, *Arachnocampa luminosa* [26], has been well studied due to the long-term tourism interest [3; 13; 27; 28].

**Fig 1:**
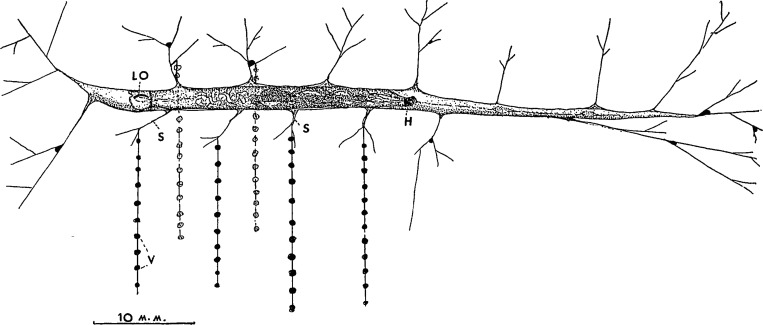
*Arachnocampa* larva within its tube, moving from left to right with its head (h). The larva attracts the prey with its light organ (LO) and then catches it with adhesive threads, made of silk (s) and adhesive vesicles (v). Image by Gatenby (1959) and reproduced with the permission of the Royal Society of New Zealand.

*Arachnocampa* has a unique silk and adhesive system used for support and prey capture. Upon hatching, the larvae construct a nest composed of a mucous tube (up to 40 cm long) in which they lie suspended from the substratum (Fig 1) [29]. Long threads (up to 105 in number; also named “fishing lines”) [30; 31] with evenly-spaced adhesive droplets (≈ ∅ 1 mm) are vertically attached to the mucous tube to form an adhesive curtain [16]. The fishing lines are between 1 and 50 cm in length and are spaced 5 mm apart [16]. These threads, composed of cross-β-sheet-rich silk [32; 33], have a similar function to spider webs in trapping flying insects [34; 35]. Prey items that have been caught in the fishing lines are pulled up by the larva using its mouthparts to repeatedly grab and haul up the thread on which the prey has been stuck. The principal food of *A*. *luminosa* in the Spellbound Cave, a part of the Waitomo Caves system, is the midge *Anatopynia debilis* (= *Tanypus debilis*, Fam. Chironomidae) [36; 37], which breeds and emerges in the mud banks and streams within the caves (unpublished observation by the first author). Nevertheless, it has been established that *A*. *luminosa* eats every type of prey caught in the fishing lines, including flying insects (moths, mayflies, sand- & stoneflies, or other *A*. *luminosa* individuals) or crawling insects (isopods, ants, amphipods, millipedes, or even small land snails) [16; 30; 31; 38; 39]. After eating, *A*. *luminosa* always removes the remains of its meal from the nest, so that the fishing-lines are kept clean for further prey capture [31]. In the past, it was assumed that glowworms used a poison (oxalic acid) in their snares [40; 41], but later analysis rejected this hypothesis [37].

**Fig 2:**
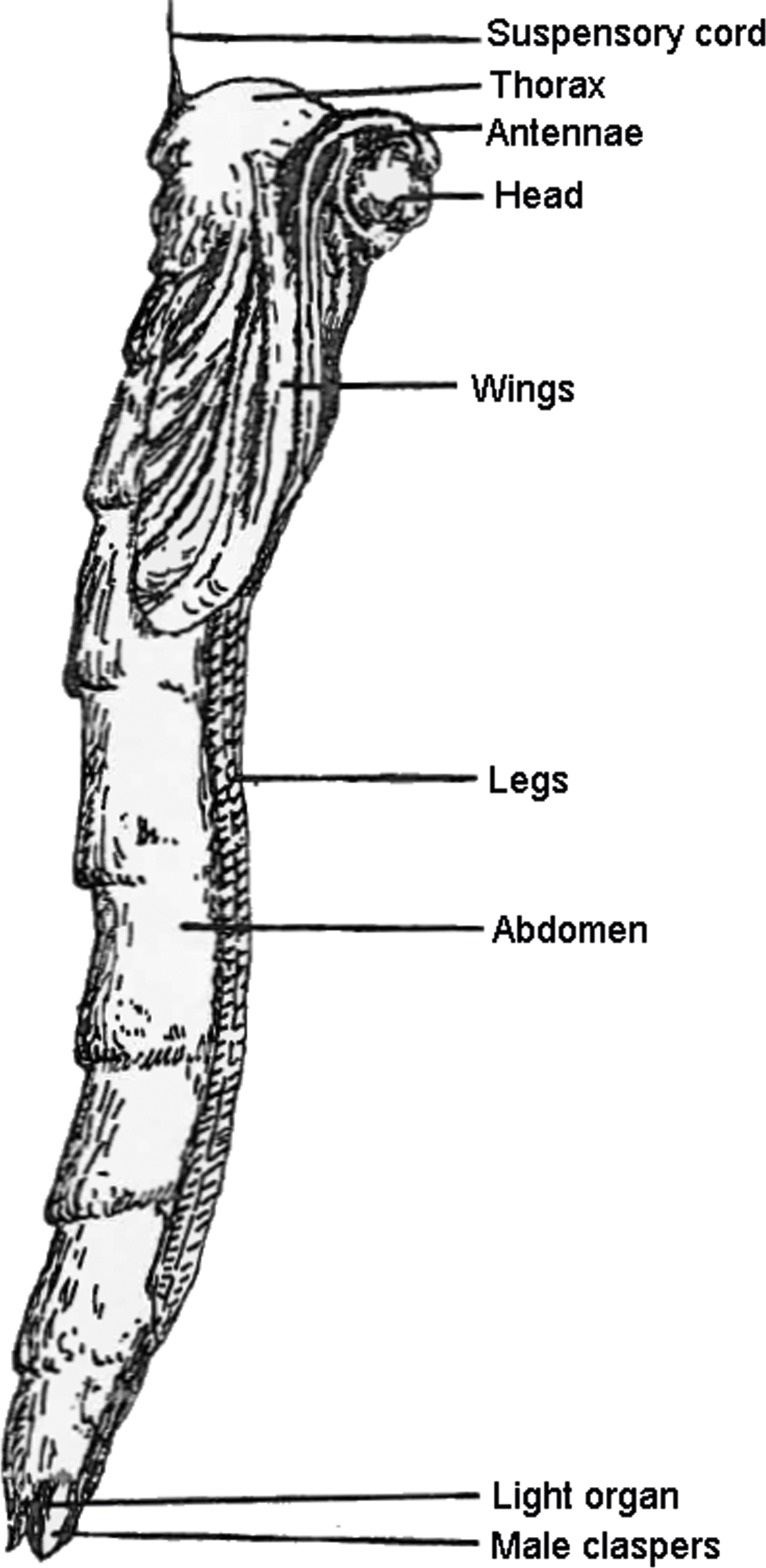
The pupa of *A*. *luminosa* spins a suspensory cord from the thorax to the cave ceiling before pupation. Image by 64 and reproduced with the permission of the New Zealand Speleological Society (NZSS).

In any one species, the length of the larval lines is known to vary depending on the habitat and degree of wind disturbance: they tend to be shorter in forests and wind-prone areas and are longer in still, moist environments such as caves [42; 43]. Before pupating, the larva removes the long fishing-lines and braces them from the snare [16]. A "defensive" circle of shorter, condensed fishing lines is left with a gap of about 20 mm on either side of the larva [16; 29; 34]. The larva then shrinks and becomes translucent, after which the New Zealand species, *A*. *luminosa*, suspends itself vertically via a single filament attached from the substratum to its thorax (Fig 2). In Australian species, the pupa is suspended horizontally via two filaments [44].

As known for caterpillars such as *Bombyx mori* or raspy crickets (*Apotrechus illawarra* and Hyalogryllacris species), *Arachnocampa* secretes the silk through labial glands in the mouthparts [1; 32] and not from spinneret glands located at the tip of the abdomen as in spiders and some insects [32; 35; 45; 46; 47]. Nevertheless, the threads of *A*. *luminosa*, with their regular arrangement of adhesive droplets, appear to be structurally similar to the sticky prey-capture threads in orb-webs [48; 49; 50; 51; 52].

Within the present study, we aim to characterize for the first time the morphology of the fishing lines in different *Arachnocampa* species and to shed light on the composition of the salivary secretion droplets. The data will show whether the threads are really “spider-like”, as suggested by the name component “*Arachno*”, or differ completely in structure and composition to those of spiders.

## Material and Methods

For the planned investigations, specimens of *Arachnocampa luminosa* and their threads were collected from October to November 2014 by J. von Byern, V. Dorrer and P. Chandler in Spellbound Cave and in Hollow Hill Cave (both in the Waitomo District, North Island, New Zealand). For comparison, individuals of the Australian species *A*. *tasmaniensis* were provided by D. Merritt (Queensland, Australia) and samples of *A*. *richardsae *were collected by J. von Byern December 2014 in the Newnes Glow Worm Tunnel (New South Wales, Australia).

### Cave descriptions

In the past, most studies of glowworm ecology and bioluminescence have been made in the Waitomo Glowworm cave, where the climate and glowworm habitat is well described [37; 38]. In the present study, we choose two different caves, Spellbound and Hollow Hill, as the larvae were more accessible without disturbing tourism operations.

Spellbound Cave (S38° 19´549; E 175° 04´454) is a commercial tourist cave with a wide and easily-accessible entrance and a large open stream named Mangawhitikau (manga = stream, whiti = to cross, kau = no) passing through the cave. The glowworms collected were situated relatively close to the cave entrance (dimly illuminated during the day). During our investigations hundreds of mayflies appeared daily at dusk in the cave and approached any type of light (glowworm or artificial torch).

Hollow Hill Cave (S38° 14´268; E 175° 01´336), in contrast, is a wild cave; visitor access is limited by permit and only possible by abseiling from a narrow T-hole. The animals collected in the present study were situated deeper in the cave, above an overhang, with a strong air flow and a creek passing deeper through the cave. Mayflies and other potential prey were not observed during the collection period, however most of the collecting was performed during the day (because of the complete darkness within the cave) and not during night, as was done in the Spellbound Cave.

In both caves, the temperature was around 13–15°C during the collection period and had a constant relative humidity around 98% at the thread collection sides.

Collection permission for *A*. *luminosa* from the Spellbound Cave was given to the first author by Mr. P. Chandler (Manager of the Spellbound Cave) and by Mr. Smith and Ms. Hayward from the Department of Conservation (39535-RES), for the *A*. *richardsae* by Mr Stone (National Parks and Wildlife Service, Blue Mountains Region) and by Mr. Cox (Biodiversity and Wildlife Team, National Parks and Wildlife Service, New South Wales, Australia) (Scientific Licence Application SL101480) and for *A*. *tasmaniensis* to the co-author Dave Merritt (FA15014).

For the **standard scanning electron microscope** (SEM) analyses the following samples were collected:

**a). Threads** of all three *Arachnaocampa* species were pulled from the nests and directly attached to aluminium stubs or hydrophobic glass slides and air-dried or dried in a desiccation chamber (filled with the moisture absorber product DampRid™, consisting of calcium chloride powder) and kept at room temperature. These samples were also used for elemental analyses (see below). Some threads were individually collected from the nest, attached vertically to a metal cage and either air-dried or dried in the desiccation chamber before being attached to the stub.

**b). Pupal suspensory cords** left behind after eclosion of *A*. *luminosa* adults were detached from the rocky substratum and fixed in 2.5% glutaraldehyde in a 0.1 M sodium-cacodylate buffer (pH 7.4) for 2 h at 25°C. Images of the cords were taken with a stereomicroscope (Model Nikon SMZ 25) followed by processing for SEM. They were washed three times in buffer solution, then distilled water, then dehydrated in a graded ethanol series, then washed several times in 100% acetone and dried with HMDS (Hexamethyldisilazane).

Samples were mounted with doubled-sided carbon tape on the stubs, coated with gold in a sputter coater (Mod. Bal-Tec SCD005) and viewed using a Jeol IT 300 SEM.

For **cryo-SEM** studies, some fishing lines were deposited vertically on pre-cooled aluminium stubs and immediately frozen in liquid nitrogen. Further analyses took place in the SEM (Mod. Jeol IT 300), equipped with the cryo-preparation device GATAN ALTO 2500 or an FEI Helios 600 Dualbeam (Hillsboro, USA) equipped with a Quorum Technologies PP2000T cryo–preparation system (East Sussex, UK).

Threads were examined using **polarized light microscopy** (Olympus AX70, Japan). Chamber-dried threads were directly attached to clean glass slides or were embedded in the mounting medium DPX (Co. Sigma Aldrich USA, Cat. No. 317616). Some were rehydrated at 100% humidity in a petri dish before examination.

For **elemental analyses** the energy dispersive X-ray spectroscopy (EDX) method (Software Team, Version 4.3, Co. Ametek Germany) was used at an acceleration voltage of 5–10 kV. The following samples were used: **A)** freshly attached **threads** of the species *Arachnocampa luminosa*, *A*. *richardsae* and *A*. *tasmaniensis* **B) water** from the Spellbound Cave ceiling, stalagmites and river passing through and **C)** specimens of the **midge *Anatopynia debilis*** from the Spellbound Cave, fixed in ethanol and homogenised. As it was proposed that the glowworm glue may contain **D) oxalic acid, urea** or **uric acid**, EDX measurements of these chemicals (Cat. No. 241172, Cat. No. U5378, Cat. No. U2625 all from Co. Sigma Aldrich USA) were likewise performed.

Samples A, B and C were directly attached or dropped onto blank aluminium stubs and air-dried, whereby sample A was additionally dried in a desiccation chamber (see description sample type B in the SEM section). The crystals of sample type D were directly attached or first diluted in water and then dropped onto the stub and dried at room temperature. Samples were not sputtered with gold or carbon, but were directly observed with a JEOL IT 300 equipped with an EDX spectrometer with a lithium—drifted silicon detector crystal. The software program EDAX Genesis 5.11 (Co. Mahwah, USA) was used for analysis. The collecting time of the elements in the samples was 100s and a ~30% dead time. Additionally, the SEM stubs of sample A were measured and photographed, after which they were extensively washed in distilled water several times and re-examined in the SEM and the elemental composition was measured.

For a **histochemical** characterization of the adhesive droplets, the threads were attached to glass slides and several histochemical stains applied (PAS; Alcian Blue 8GX, Toluidine Blue O at pH 4.3, Biebrich Scarlet at pH 6.0, 8.0, 9.5, and 10.5, Sudan black B and Arnow DOPA test) (for methodical details see [53]). Subsequently, the samples were dehydrated in ethanol, cleared in DPX and evaluated.

Elemental analyses by **X-ray Photoelectron Spectroscopy** (XPS) spectra were taken using a Kratos Ultra system applying the following acquisition parameters: base pressure: 4*10^−8^ Pa, sample neutralization applying low energy electrons (kinetic energy <5 eV), hybrid mode (electrostatic and magnetic lenses are used), take off angle of electrons 0°, pass energy 20 eV in high resolution spectra and 160 eV in survey spectra, excitation of photoelectrons by monochromatic Al K_α_ radiation. The binding energy calibration was performed by referring the C1s component of aliphatic carbon species to 285.0 eV, and a Shirley background was considered. In the case of sample No. 2, the sample was measured initially, then extensively washed with distilled water for 1 min and measured again.

### Statistical evaluation

As glowworms react very sensitively to a continuous removal of their threads, we limited the planned experiments to the necessary minimum. Moreover, we aimed to evaluate animals and nests that were similar in size and amount of threads. These limitations may have led to a high standard deviation and non-representative sample numbers in some cases. However, the well-being of this sensitive animal was paramount.

Calculations of the **number of threads** of 31 nests from Spellbound and 37 nests from Hollow Hill and **weight** of a **single thread** per nest (in total 2767 threads from the 31 nests in Spellbound and 2376 threads from the 37 nests in Hollow Hill) were performed for *A*. *luminosa* specimens only. For the analysis, the threads with their droplets were removed from the nest and the number of threads per nest counted, then all threads of each nest were grouped and weighed and the mean weight of a single thread was calculated. At Hollow Hill we were also able to collect and re-calculate the **number** (from 9 nests) and **weight of threads** from nests that remained after the adult had eclosed and departed, leaving the **pupal exuvium** behind (n = 816 threads). In the Spellbound Cave, no pupal exuvia were found during the collection time for comparison.

Estimations of the **weight** (n = 4067 droplets) and **distance** of the normal **thread droplets** (n = 192 threads) were performed with threads isolated from 43 nests from Spellbound and attached to a metal frame. The threads were photographed and the number of droplets was counted in relation to thread length. To calculate the weight of the droplets, the threads were taken off, weighed, dried and re-weighed. The threads (n = 166 threads including end-droplets) were isolated from the 47 nests from Spellbound, applied to the metal frame with the end-droplet hanging freely. The threads were photographed and the end-droplet was digitally measured. To be able to re-calculate the weight of a single end droplet, the threads were collected, weighed, dried and re-weighed, as the mass of the thread had to be subtracted from the overall mass.

For the **hammock weight** (n = 36 nests from Spellbound) the threads were removed from the nest, the larva was removed and relocated within the cave. The remaining horizontal structure comprising the hammock was removed and weighed.

For the **weight loss experiments**, all threads of a nest (n = 3) were collected, weighed and stored in pre-weighed Eppendorf tubes. The samples were subsequently dried at 20°C under 60% relative humidity or 30% relative humidity in the desiccation chamber, filled with the moisture absorber. The 60% value was chosen because higher values (70–90%) could not be kept constant over the measurement period. The 30% chosen because it is one-half of the consistent 60% humidity level. Pure water and raw *Bombyx mori* silkworm fibres [54], soaked in water for 24 h, were used as control. All samples were weighed every 12 h until no more weight loss ceased. For the statistical evaluation, the empty Eppendorf tube was defined as 100% and the daily measured weight loss was calculated for each time point.

Measurements of the **droplet size** (length x width) were performed with randomly chosen threads from two specimens. The threads were attached vertically to glass slides and air-dried. The measurement and counting of fresh threads hanging down from the nest or with photographs was inappropriate due to the bad lighting conditions within the cave. Consequently, confocal laser scanning microscope Leica (Mod. TCS SP5 DM-6000 CS) was used for evaluation and measurement. Statistical evaluation of all data was performed with the Windows software Excel and visualized as boxplot diagrams using the software OriginPro9.1.

The ability of larvae to **replace** removed **threads** was examined. Selected nests (n = 10) had all threads counted, removed and later weighed (defined as initial). These nests were marked and the procedure (thread counting, removal, weighing) was repeated 24 h, 48 h and 96 h after the initial counting.

The pupa suspensory cords were investigated with a **transmission electron microscope (TEM)**. Samples were fixed in the same fixation solution as for SEM, then washed three times for ten minutes in the buffer and post-fixed in 1% osmium tetroxide (diluted in 0.1 M cacodylate buffer at pH 7.4) for one hour. After step-wise dehydration, the samples were infiltrated and embedded in Epon epoxy resin (AGAR 100, Agar Scientific, UK). Polymerization took place at 60°C for three days. Semi-thin sections (1 μm thickness) were made with a Leica UC7 microtome (Leica Microsystems, Austria). Ultrathin sections (50–70 nm), performed with the same instrument, were mounted on copper slot grids, coated with formvar in dioxane, contrasted with uranyl acetate and lead citrate [55], and examined in a Zeiss Libra 120 TEM.

Unfortunately, we failed to provide images and cross-sections of a fishing line within this study as the thread lightness and minor wettability restricted any fixation and embedding into resin. Different approaches were tested, i.e. spanning the threads in a u-shaped profile and immersing them in fixative solution (incl. pure osmium tetroxide) or fixative-gassing them for days in a tight glass cylinder within the cave; embedding the dried threads, attached to aclar foil, in resin; direct immersion of the threads in liquid resin or winding the threads onto Eppendorf tips and then continuing as for the pupa suspensory cords.

## Results

### Snare morphology of *Arachnocampa luminosa*

**Fig 3:**
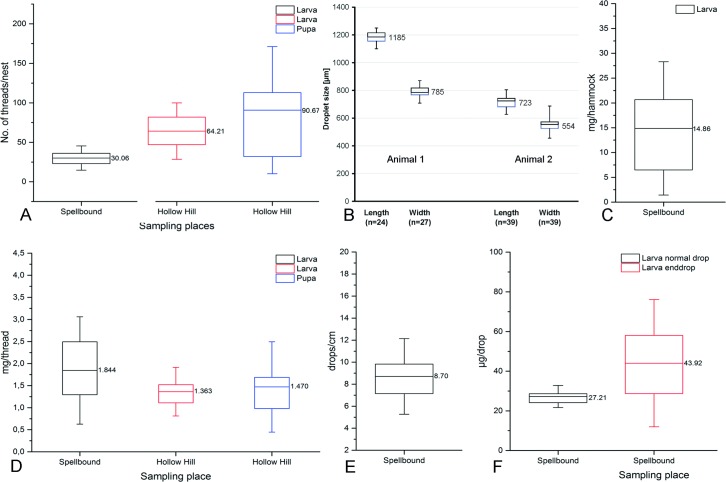
Evaluation of the New Zealand species *A*. *luminosa* indicates that **A)** the larva in the Spellbound Cave produce fewer threads (mean 30 per nest) in relation to those from Hollow Hill cave (mean 64 per nest) or the pupae (mean 90 per nest) from Hollow Hill. **B)** Droplets attached to glass slides have a greater length than width and vary between individuals, e.g. 1185x785 μm (length x width) in animal 1and 723x554 μm in animal 2.**C)** The hammock (excluding threads and larva) from animals in the Spellbound Cave with a mean weight of 14.86 mg. Weight measurements with samples from the Hollow Hill cave could not be performed, as it is forbidden to remove a larva from its nest. **D)** Threads taken from the nest are much heavier (in mean 1.844 mg/thread) in the Spellbound Cave than those from Hollow Hill (1.36 mg) or produced by the pupae (1.47 mg). **E)** On average, the larva produce around 8 droplets per cm thread length, with **F)** a estimated weight of 27.21 μg for the normal droplet and almost 44 μg for the final droplet at the thread tip.

In Spellbound, the larvae produced around 30 threads per nest (n = 31 nests), while in Hollow Hill the larvae produced 64 threads (n = 37 nests) on average (Fig 3A). The threads (including droplets) of those from Spellbound were heavier (≈ 1.8 mg) in relation to those from Hollow Hill (≈ 1.4 mg) (Fig 3D).

It was observed during collection that the threads from Hollow Hill were longer than those at Spellbound. Differences in the number and length of the threads were also related to the size of the larva, with larger specimens (> 3 cm length) having longer threads than smaller (< 3 cm length) individuals (not measured).

In Hollow Hill we observed that pupae possessed about 90 short threads (Fig 3A), circularly arranged around the animal. The short threads surrounding the pupae were similar in weight (≈1.5 mg) to those produced by the larvae (≈1.4 mg) (Fig 3D). Large *A*. *luminosa* larvae (> 3 cm) weighed around 101 mg and medium-sized larvae (≈ 2 cm) were between 28 and 35 mg.

### Characterization of the adhesive droplets

#### Macroscopic level

**Fig 4:**
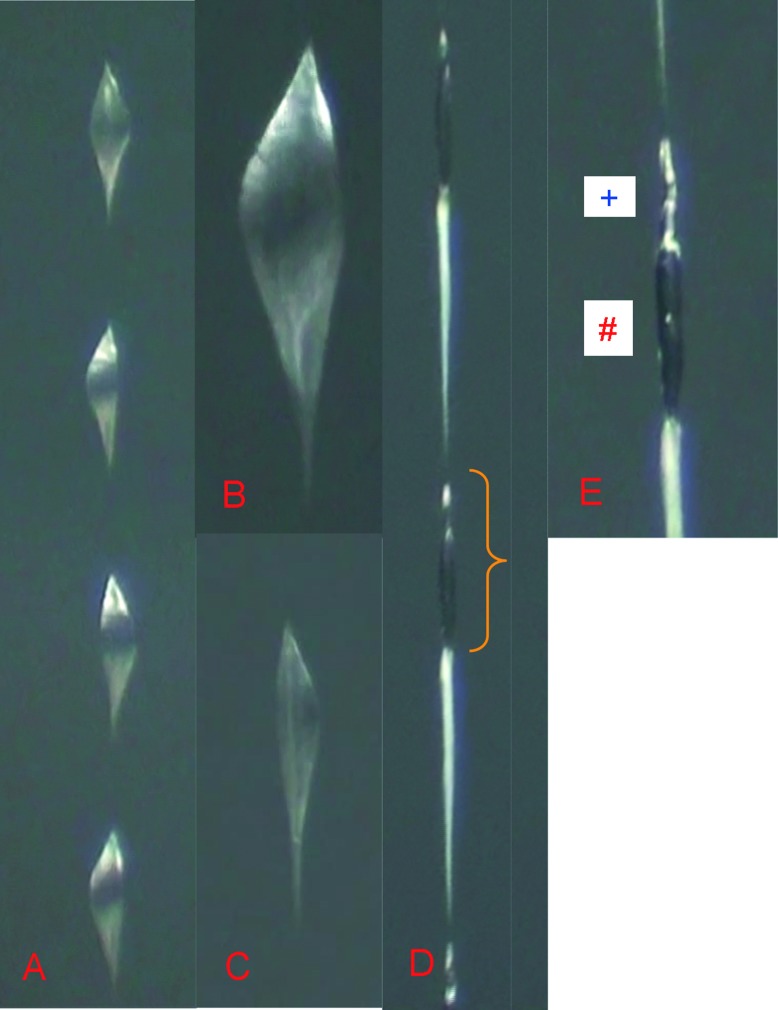
The adhesive droplets of the threads in *A*. *luminosa* are **A)** evenly distributed and **B)** ellipsoid shaped, with a **C)** central orientated thread. Maintaining the thread at a humidity of 30% results in the disappearance of the droplet at the macro level, only the **D)** thread and the droplet area remain visible. At a higher magnification (see yellow bracket) **E)** two evenly-spaced regions could be identified, an upper one (blue plus) with a shiny metallic appearance and a lower, dark region (red hash).

**Fig 5:**
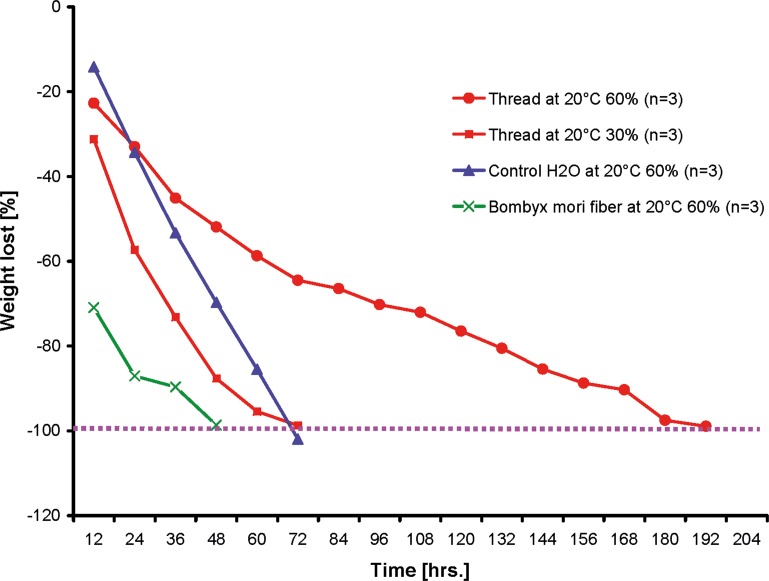
Under conditions of high humidity (60%), a low water loss over time (8 days) was determined (red circled line), while at a lower humidity (30%, red squared line) there was a fast weight loss (after 3 days). In relation to this, water (blue line) showed a more constant evaporation rate at 60% humidity and likewise reached its final minima after 3 days. *Bombyx mori* silkworm fibres (green line), watered for 24 h, evaporated within 2 days, showing the highest weight loss within the first 12 h.

The adhesive droplets were evenly spaced along a silk thread of *A*. *luminosa* (Fig 4A), with a mean of 8.7 droplets per mm (around ≈125 μm between the droplets) (Fig 5E). The droplets did not have a tear-drop form, possessing instead a pointed ellipsoid shape (Fig 4B and 4C) oriented along the central vertical thread (Fig 4C). The dimensions of the droplets in *A*. *luminosa* varied between specimens, from 1185 x 785 μm (mean length x width size) (Fig 3B) in animal 1 to 723 x 554 μm in animal 2, and the droplets had a mean weight of 27 μg for the normal thread droplet along a silk thread and 44 μg for the final droplet at the thread tip (Fig 3F). The isolated hammock (excluding the threads and larva) had a mean weight of ≈15 mg (Fig 3C).

Maintaining the isolated threads at a low humidity level (30%) in a desiccation chamber resulted in a 98% weight loss of within 3 days (Fig 5). Furthermore, the droplet disappeared completely in this state at a macroscopic level; only the thread and the droplet’s outer shape remained visible at the microscopic level (Fig 4D). Higher magnification of this “dried” fishing line (Fig 4E) showed that the thread itself was relatively wide below the droplet area and tapered uniformly to the next droplet. In the dried droplet, two regions could be identified, an upper region with a shiny metallic appearance and a lower, dark region. At higher humidity (60%) the threads took longer (up to eight days) to completely dry. Under both 30% and 60% relative humidity, the highest weight loss was observed within the first 12 h (-31% at 30% humidity and -22% at 60% humidity). In contrast, weight loss in the control water sample (1 ml water in an Eppendorf tube, at a volume almost equal to that of the glue sample) was initially lower (-14% after 12 h) and was complete after 3 days, similar to the thread sample at 30% humidity. However, water loss was more continuous in relation to the weight loss of the *A*. *luminosa* thread. By comparison, the *Bombyx mori* silkworm fibres exhibited a high weight loss after 12 h (-70%) and were completely dry after 48 h.

#### Microscopic level

**Fig 6:**
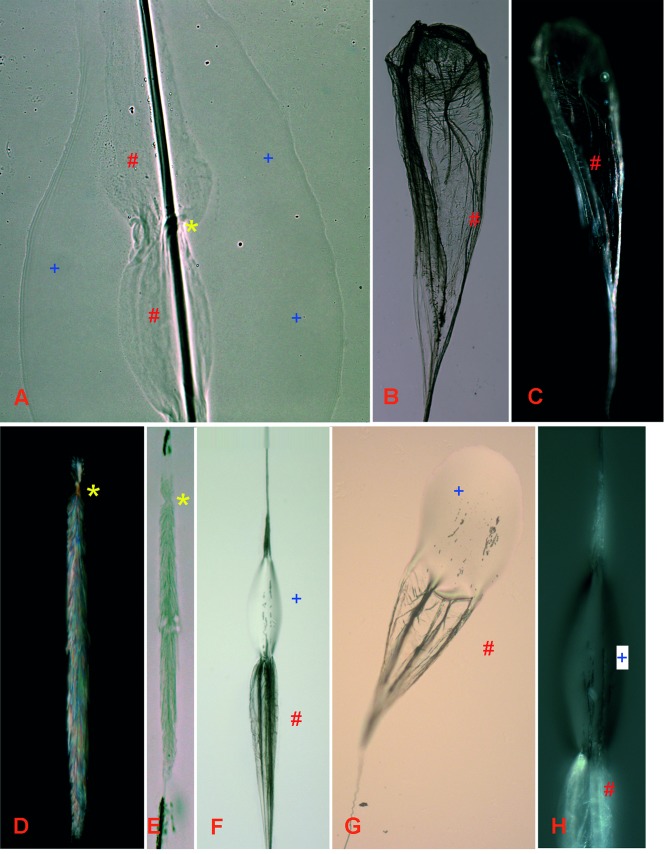
When maintained at 30% humidity, **A)** the water droplet contour (blue plus), as well as the core (red hashtag) of the adhesive thread remain visible. Attachment of the core to the thread takes place in the central part of the droplet (yellow asterisk), the core is constricted and encloses the thread in this area. **B)** In the dried state, the core (red hashtag) of the final droplet mostly appears as a web-like ballooning structure and is **C)** optically active in the polarized microscope. Also, the core of the normal thread droplet appears crystalline in the dried state, being **D)** optically active in the polarized microscope **E)** and reflective in the light microscope. Under highly humid conditions (100%), a re-hydration (blue plus) of the core (red hashtag) for the **F)** normal droplet as well as the **G)** final droplet takes place. **H)** In the polarized microscope the re-hydrated part of the core (blue plus) is optically inactive in relation to the dried area (red hash).

**Fig 7:**
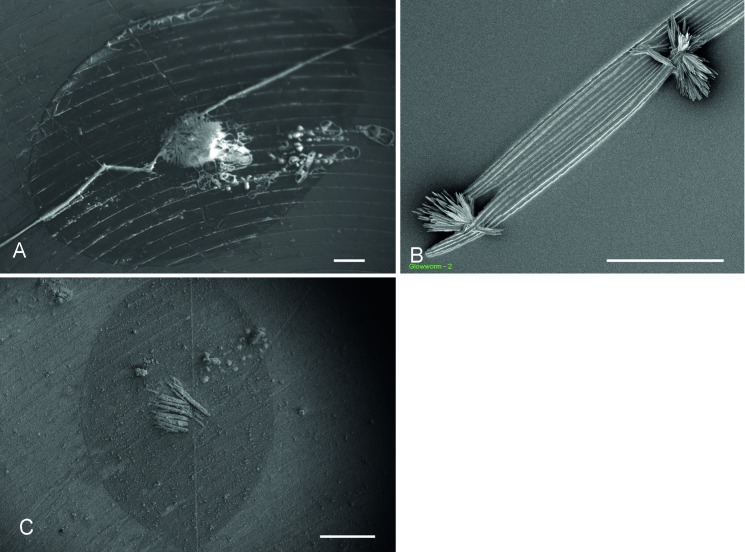
**A)** and **C)** Cryo-fixed threads are almost roundish and there is crystal formation within the water droplet area during sublimation. **B)** Threads collected on hydrophobic glass slides appear partly flat, with incorporated crystals between the fibres. Scale bar in A = 100 μm; B = 20 μm; C = 200 μm.

Despite the disappearance of the droplet at the macroscopic level, at the light microscope level the droplet contour remained visible, even when dried at 30% humidity conditions with the moisture absorber powder (Fig 6A). After slide-mounting in a water-insoluble medium such as DPX, the droplet contour disappeared and only its core and the thread itself remained visible. In this dried state, the core of the final droplet had the appearance of a web-like ballooning structure (Fig 6B) and showed birefringence in the polarized microscope (Fig 7C). The core of the normal thread droplet had the appearance of a compact, crystalline coating and showed birefringence in the polarized microscope (Fig 6D) and reflectivity in the light microscope (Fig 6E). An attachment/cross-over point at the core of the thread could be observed in the polarized as well as the light microscope (yellow asterisk in Fig 6A, 6D and 6E). The thread itself showed no birefringence in the polarized microscope.

Exposure of a dried thread attached to a glass-slide for several minutes at 100% humidity resulted in a re-hydration of the droplet, starting centrally at the core in the case of the normal droplets (Fig 6F) and at the thread tip point in the case of the final droplet (Fig 6G). In this re-hydrated area, the crystal structure of the core showed birefringence (Fig 6H) in the polarized microscope. This process of de- and re-hydration could be repeated several times.

None of the histochemical stains tested (PAS; Alcian Blue 8GX, Toluidine Blue O, Biebrich Scarlet, Sudan black B and Arnow DOPA) showed any reaction to the thread or droplet, indicating an absence of carbohydrates, lipids or proteins (acidic or basic) in the *Arachnocampa* glue.

#### Scanning Electron microscopy

**Fig 8:**
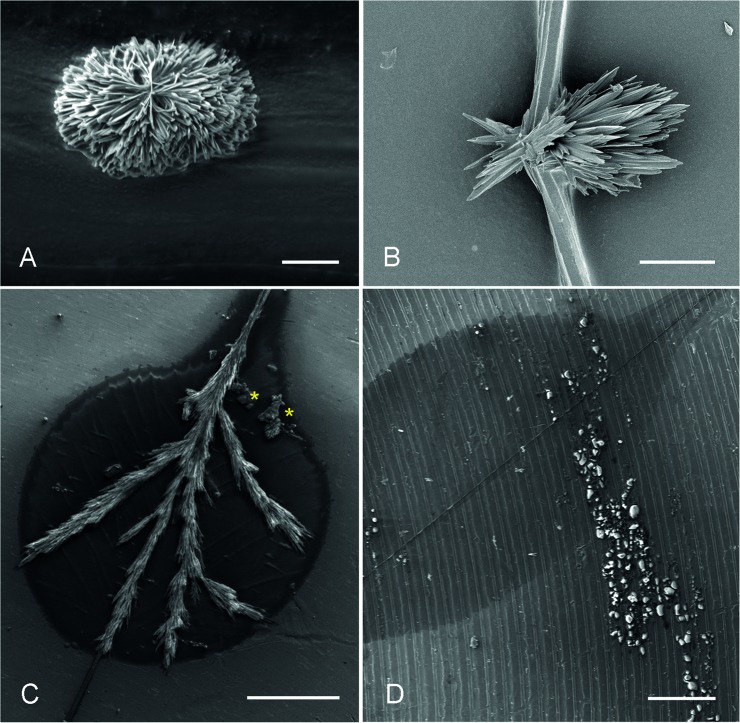
In the scanning electron microscope the crystalin-like core of the normal thread droplet in *A*. *luminosa* appears **A)** as rosettes or **B)** sheet-shaped pointed bundle, when pre-dried in the desiccation chamber before attachment on stubs. **C)** Samples, attached to polished aluminum stubs and subsequently dried in the desiccation chamber; the crystals grow needle-shaped along the thread and form lateral branches. In addition to the crystal at the centre of the droplet, a non-crystalline content (yellow asterisks) could be observed at the base of the droplet. **D)** Samples, freshly attached to a riffled stub and air-dried; no crystal formation within the droplet area could be observed. Scale bar in A = 10 μm; B = 5 μm; C and D = 200 μm.

In the cryo-SEM, the threads either appeared almost roundish in the frozen stage (diameter around 8 μm) (Fig 7A) or flat with several parallel fibres and incorporated crystals (Fig 7B) when dried on hydrophobic glass slides. During sublimation of the frozen thread in the cryo-SEM, a crystal formation within the water droplet area was observed (Fig 7C). Pre-dried threads showed the presence of irregular crystals in the droplet area, arranged in the form of rosettes (Fig 8A) or sheet-shaped pointed bundles (Fig 8B) around 10–30 μm long. No difference in crystal formation could be observed in air-dried or chamber-dried fishing lines. In samples that were freshly attached to a polished stub and then dried in the desiccation chamber, the crystals within the droplet area were needle-shaped and oriented along the thread with lateral branches (Fig 8C). The position and size of the adhesive droplets were visible as an outline on the mounting stub. In addition to the crystal at the centre of the droplet, a non-crystalline content could also be observed which represented the outline of the droplet. In samples that were freshly attached to a riffled stub and then air-dried, no crystals were present within the droplet area (Fig 8D). Instead, the droplet content was lined along the stub riffle and appeared as granular material similar to that in Fig 8C. Again, the contour of the original adhesive droplet and the central fishing line was clearly visible.

No differences in crystal formation, morphology or size could be observed between the three *Arachnocampa* species.

### Elemental analyses of the thread and droplets in *Arachnocampa* species

**Fig 9:**
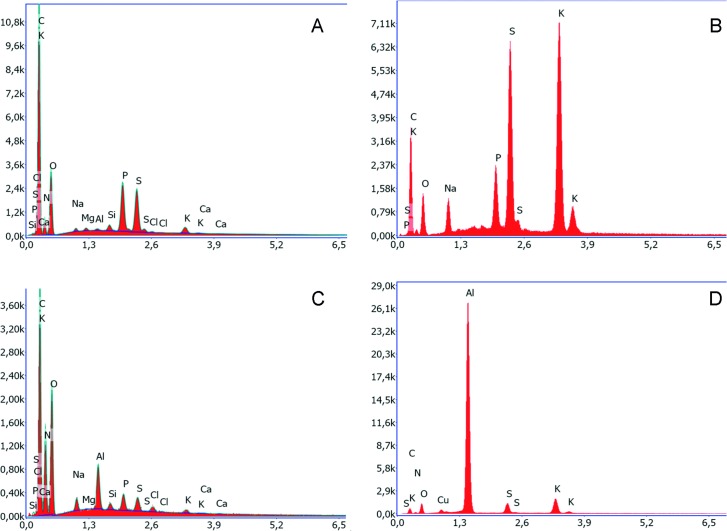
Elemental analyses indicate a certain amount of **A)** phosphorus and sulphur in the thread of *A*. *luminosa* while **B)** in the chamber-dried crystals a high proportion of sodium, phosphorus, sulphur and potassium could be measured. **C)** In relation to this, in the chamber-dried crystals of *A*. *richardsae* there is a small proportion of phosphorus, sulphur, chloride. **D)** In the chamber-dried crystals of *A*. *tasmaniensis* there is only a small proportion of sulphur and potassium.

The **thread** of ***A*. *luminosa*** mainly consisted of the elements carbon and oxygen and a smaller amount of nitrogen (Table 1, Fig 9). The trace elements sodium, phosphorus, sulphur and potassium were detectable, while elements such as calcium, chloride and magnesium were below the detection limit of 0.1%. In the chamber-dried **crystal** sample of *A*. *luminosa* similar values were given for some elements (carbon, nitrogen, oxygen, magnesium, phosphorus and chloride). It could not be excluded that these values corresponded to the thread below the crystal, due to the assumed electron penetration depth of ≈ 1–2 μm. Other measured elements, such as sodium, sulphur and potassium, were probably related to the crystal sample type as they constituted a low percentage in the thread sample. Slightly lower values were given for air-dried samples; also, these crystals were more widely distributed on the stub than the chamber-dried crystals.

**Table 1:** EDX values of the different elements and chemicals, given in atomic%, present in the different glowworm species.

	*A*. *luminosa*			*A*. *richardsae*		*A*. *tasmaniensis*	
Elements *	Thread [atom %]	Crystal Chamber-dried [atom %]	Crystal Air-dried [atom %]	Thread [atom %]	Crystal Chamber-dried [atom %]	Thread [atom %]	Crystal Chamber-dried [atom %]
**Carbon**	27–29	20–29	27–29	52–55	3–11	50–52	24–31
**Nitrogen**	3–4	≤ 4	6–8	20–22	64–68	15–17	4–9
**Oxygen**	67–68	64–66	47–49	20–22	0.6–0.8	19–21	30–32
**Sodium**	0.1–0.4	1.6–2.0	0.2–0.4	2–3	0.01–0.1	1.4–1.8	≤ 0.4
**Magnesium**	- 0.06	≤ 0.04	0.06–1.4	0.1–0.2	0.2–0.5	0.2–0.4	
**Phosphorus**	0.6–1	0.6–1.1	≤ 0.03	1.5–2	0.1–0.3	1–1.5	
**Sulphur**	0.2–0.6	1.5–3.0	1–1.6	0.2–0.3	≤ 0.1	0.15–3	1.5–2.5
**Chloride**	≤ 0.02	≤ 0.03	≤ 0.33		≤ 0.1	0.2–0.3	0.1–1
**Potassium**	≤ 0.2	1.4–4	0.2–0.5		≤ 0.1	0.5–0.7	2–3.5
**Calcium**			≤ 0.1			1.5–1.6	

*excluding elements (Aluminium, Silicium, Copper) present in the stubs

The thread and crystal composition of ***A*. *richardsae*** differed from *A*. *luminosa*. In the Australian species, the **thread** contained a higher quantity of carbon, nitrogen, sodium and phosphorus, while a much low oxygen value was measured in the thread. Other elements, such as magnesium and sulphur, were present at similar levels. There were also differences in the elemental composition for the **crystal** samples: In *A*. *richardsae* an increased nitrogen and magnesium amount was measured, while the carbon, oxygen, phosphorus, sodium, sulphur and potassium levels were low.

The elemental composition of the **thread** in ***A*. *tasmaniensis*** was close to those of *A*. *richardsae*, having a high amount of carbon and a similar level of nitrogen, oxygen, sodium, magnesium, phosphorus. However, the thread of *A*. *tasmaniensis* contained a higher amount of sulphur and calcium, both of which were not present in the threads of the other two species. In contrast to the thread correlation of *A*. *richardsae*, the **crystals** of *A*. *tasmaniensis* resembled those of *A*. *luminosa*, having a high value of carbon, oxygen, sulphur and potassium but a low amount of nitrogen. Furthermore, the crystals lacked phosphorus, which was present in *A*. *luminosa* and *A*. *richardsae*, but, in contrast to the others, contained a high amount of chloride.

Extensive washing of the chamber-dried threads/crystals (all three *Arachnocampa* species) attached to SEM stubs with distilled water did not lead to a change in the crystal structure or elemental composition of the threads and crystals.

Both the **river** passing through the Spellbound Cave and the **water** from the **cave ceiling** and **stalagmites** mainly contained the elements oxygen and calcium (Table 2) and, to a lesser extent, sodium. Nevertheless, there were differences between the cave ceiling and the other two samples (river and stalagmite). While the ceiling sample bore a high level of chloride and a low value of carbon, opposite rates were given for the other two samples. In the stalagmite and river water, there were traces of magnesium and sulphur, both of which were lacking in the cave ceiling sample. Only in the river water could a small amount of potassium be measured.

**Table 2:** EDX values of the different elements and chemicals, given in atomic%, present in the water within the Spellbound cave.

	Water sample			Midge
Elements *	Cave ceiling [atom %]	Stalagmite [atom %]	River [atom %]	[atom %]
**Carbon**	5–7.5	9–21	18–64	59–61
**Nitrogen**				15–17
**Oxygen**	44–58	54–64	29–61	20–24
**Sodium**	0.2–1.0	0.2–0.5	0.1–1.4	0.1–0.5
**Magnesium**		0.1–0.8	≤ 0.1	0.07–0.2
**Phosphorus**				0.1–1.5
**Sulphur**		0.5	0.1–0.3	0.2–0.8
**Chloride**	7–17	0.3–1.3	0.1–1.2	
**Potassium**			0.15	0.07–0.7
**Calcium**	4–19	13–23	16–21	≤ 0.4

*excluding elements (Aluminium, Silicium, Copper) present in the stubs

In the extract of the **midge *Anatopynia debilis*** mainly the basic elements carbon, nitrogen and oxygen were detected. All the other detected elements, except chloride, were also present (Table 2).

The analyses of the **oxalic acid**, **urea** and **uric acid** chemicals indicated slight ratio differences between pure crystals and those diluted with water for some samples (Table 3): Regarding oxalic acid (chemical formula C_2_H_2_O_4_), only the elements carbon and oxygen could be measured, however this was slightly higher in the distilled water diluted sample in relation to the pure crystal type. In the urea sample (chemical formula CH_4_N_2_O), there was a minimal lower amount of oxygen in the pure crystal in relation to the diluted sample. In contrast, the nitrogen level was higher in the pure sample type, while less difference could be observed in the carbon ratio for both sample types. In the uric acid sample (chemical formula C_5_H_4_N_4_O_3_), only a slightly lower amount of oxygen and a higher amount of carbon could be observed for the pure sample type, while there was less difference between both sample types for nitrogen.

**Table 3:** EDX values of the different elements and chemicals, given in atomic%, typically produced by insects.

	Oxalic acid		Urea		Uric acid	
Elements *	pure [atom %]	Diluted [atom %]	Pure [atom %]	Diluted [atom %]	Pure [atom %]	Diluted [atom %]
**Carbon**	40–45	37–39	24–27	25–29	34–38	33–35
**Nitrogen**			47–49	34–42.5	37–41	38–39
**Oxygen**	50–56	59–62	25–29	27–37	21–28	25–28
**Sodium**	-					
**Magnesium**						
**Phosphorus**						
**Sulphur**						
**Chloride**						
**Potassium**						
**Calcium**						

*excluding elements (Aluminium, Silicium, Copper) present in the stubs

### XPS analyses of the fishing lines of *Arachnocampa luminosa*

As the surface of a biopolymer determines its adhesive and environmental interactions, the composition of the topmost 0.01 μm of the dry fishing lines was determined. Two droplet samples (No. 1 and 2), supported on gold-coated substratum, were investigated.

**Fig 10:**
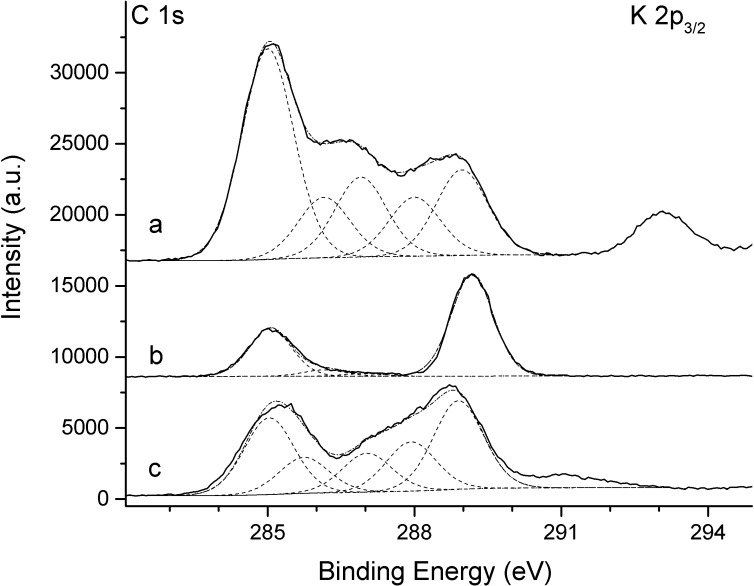
Normalised C1s signal as obtained by XPS for the fishing lines of droplet sample 1 (a), urea powder (b), and uric acid powder (c).

The results obtained, assuming a homogeneous composition of the investigated surface region, are presented in Table 4. Generally speaking, the surface of the fishing lines was dominated by the presence of organic species. Moreover, cationic sodium, potassium, and calcium species and oxo-complex anionic sulphur and phosphorus as well as silicon were detected with potassium and calcium in sample 2. Concerning the high concentration of around 14 at% to 19 at% of nitrogen-containing amounts, found for all investigated surface regions, their chemical nature was investigated in greater detail. Given a maximum concentration of predominantly monovalent cations, a prevailing anionic nature of the nitrogen-containing species was ruled out. Moreover, the N1s binding energy of 399.7+-0.2 eV indicates that the nitrogen atoms were not related to oxygen atoms and were organic in nature. An analysis of the C1s signal region shown in Fig 10 for the fishing lines of droplet No. 1 revealed several signal contributions. In detail, the peak fitting indicates aliphatic carbon species (binding energy 285.0 eV), two equally concentrated carbon species related to peptide groups (C*-N with a binding energy 286.0 +- 0.1 eV, and OC*-N with a binding energy 288.0 +- 0.1 eV), carbon C*-O in alcohol or ether groups (binding energy 286.9 +- 0.1 eV), and, finally, a pronounced signal contribution with a binding energy of 289.0 +- 0.1 eV the surface concentration of which corresponds to 7.9 at%, related to at least trisubstituted carbon atoms. Accordingly, the concentration of peptide-type nitrogen species is suggested to amount to approximately 5.5 at%, which means that around 13.8 at% of N species have to be attributed to a distinct binding environment.

**Table 4:** Surface concentrations, given in atomic%, obtained by XPS investigations probing the composition within the topmost 0.01 μm of dry fishing lines in *A*. *luminosa* and a urea powder characterised for reference purposes.

Elements	Droplet 1 [atom %]	Droplet 2 [atom %]	Droplet 2 after H_2_O wash [atom %]	Urea [atom %]	Uric acid [atom %]
**Carbon**	53.5; 53.6	44.6; 45.4	64.3	37.7	50.8
**Nitrogen**	14.5; 16.5	16.2; 19.3	17.3	41.9	27.5
**Oxygen**	24.5; 25.9	27.4; 29.2	16.3	20.4	21.1
**Silicium**	1.6	1.7; 2.0	1.3	-	-
**Sodium**	1; 1.1	1; 2	-	-	0.6
**Magnesium**	-	-	-	-	-
**Phosphorus**	2.4; 2.7	3.4; 3.8	0.4	-	-
**Sulphur**	0.5; 0.6	0.4; 0.5	0.3	-	-
**Chloride**	-	-	-	-	-
**Potassium**	-	0.8; 1.2	-	-	-
**Calcium**	-	0.3; 0.8	-	-	-

For reference purposes, a urea powder and a uric acid powder sample were examined since these organic substances represent nitrogen-rich molecules which might be related to the metabolism of *A*. *luminosa* and, following their stoichiometry of CH_4_ON_2_ and C_5_H_4_O_3_N_4_, respectively, might account for the remarkably high nitrogen concentrations found at the surface of the fishing lines investigated by XPS. Both powder samples showed centrosymmetric N1s signals, with binding energy values of 399.7+-0.1eV in case of urea, similar to the value observed for the fishing line, and 400.4+-0.1eV in the case of uric acid. The measured [N]/[O] concentration ratios of 2.1 for urea and 1.3 for uric acid corresponded to the stoichiometric values of 2 and 1.33, respectively, as expected for these materials. The evaluation of the C1s, O1s and N1s signals of the three substances is presented in Table 3, and the C1s spectra are shown in Fig 10.

The hydrocarbonaceous nature of the lowest C1s binding energy values detected in the urea and uric acid powders were attributed to ubiquitous carbon and were used to reference the binding energy values of the measured signals. Concerning the additional C1s signals of characteristic functionalised carbon atoms, the peak fitting was oriented by urea molecules showing carbon atoms in one binding environment, and by uric acid molecules showing C atoms in roughly four distinct binding environments. Correspondingly, as shown in Table 2, one C, N, or O atom corresponded to around 22+-2 at% of the material in the case of urea, and around 7.5+-1 at% in the case of uric acid. As may be inferred from Table 5 and Fig 10, the signal of the functional carbon atom C* in N-C*O-N contributed to the C1s spectrum in a region around a binding energy value of 289.0+-0.1 eV, where a signal contribution was also detected for the fishing lines of *Arachnocampa luminosa*. If the urea molecule were the binding environment for the 13.8at% of N species which were to be assigned to molecular groups, then a signal intensity of around 7 at% of N-C*O-N would be expected, in comparison to the 7.9 at% of the C species detected in the fishing lines. In contrast, the C1s spectrum of uric acid revealed a broad signal contribution of around 291 eV, which was detected neither in the fishing lines nor in urea. This signal contribution was ascribed to electronic transitions of π-electrons in the aromatic ring system of uric acid which constitutes a heterocyclic and bicyclic purine derivative. Such shake-up spectral features are known for aromatic rings [56], and they have been reported for purine systems such as poly adenosine [57].

**Table 5:** Surface concentrations, given in atomic% and binding energies (B.E.) of peaks given in eV, as related to XPS signal contributions obtained by fitting C1s and O1s XPS spectra obtained for dry fishing lines, and for urea and uric acid powders.

Peak (B.E./eV)	C1s (285.0)	C1s (286.0)	C1s (286.9)	C1s (288.0)	C1s (289.0)	C1s (291.0)	O1s (531.4)	O1s (532.7)	N1s (400)
Droplet sample	19.3	5.5	7.3	5.4	7.9	-	20.1	7.3	19.3
Urea	11.2	1.5	0.6	-	23.6	-	20.5	-	42.7
Uric acid	13.5	6.3	6.8	8.6	15.5	shake-up	21.1	-	27.5

In conclusion, an assumption of the presence of urea in the fishing lines would be in good accordance with the binding energy of the N1s signal, with the contributions to the spectral composition and even with the relative concentrations of the corresponding N and functional C atoms. However, an assumption of the presence of uric acid in the fishing lines would not be in good accordance with the binding energy of the N1s signal or the actual absence of shake-up structures in the C1s spectrum of the fishing lines.

Therefore, the presence of urea molecules on the surface of the fishing lines is strongly indicated. In the case of droplet No. 2, the XPS results obtained after washing indicated that the sodium, potassium and calcium as well as the phosphorus-based elements are, on the one hand, soluble in water and, on the other hand, situated at the upper regions of the fishing line. However, the concentration of urea was barely reduced after water contact in the droplet sample. This finding indicates that urea was also present in the volume region of the fishing lines, possibly strongly attached to peptide moieties, as suggested by [58].

### Structure of the Arachnocampa luminosa pupa suspensory cord

**Fig 11:**
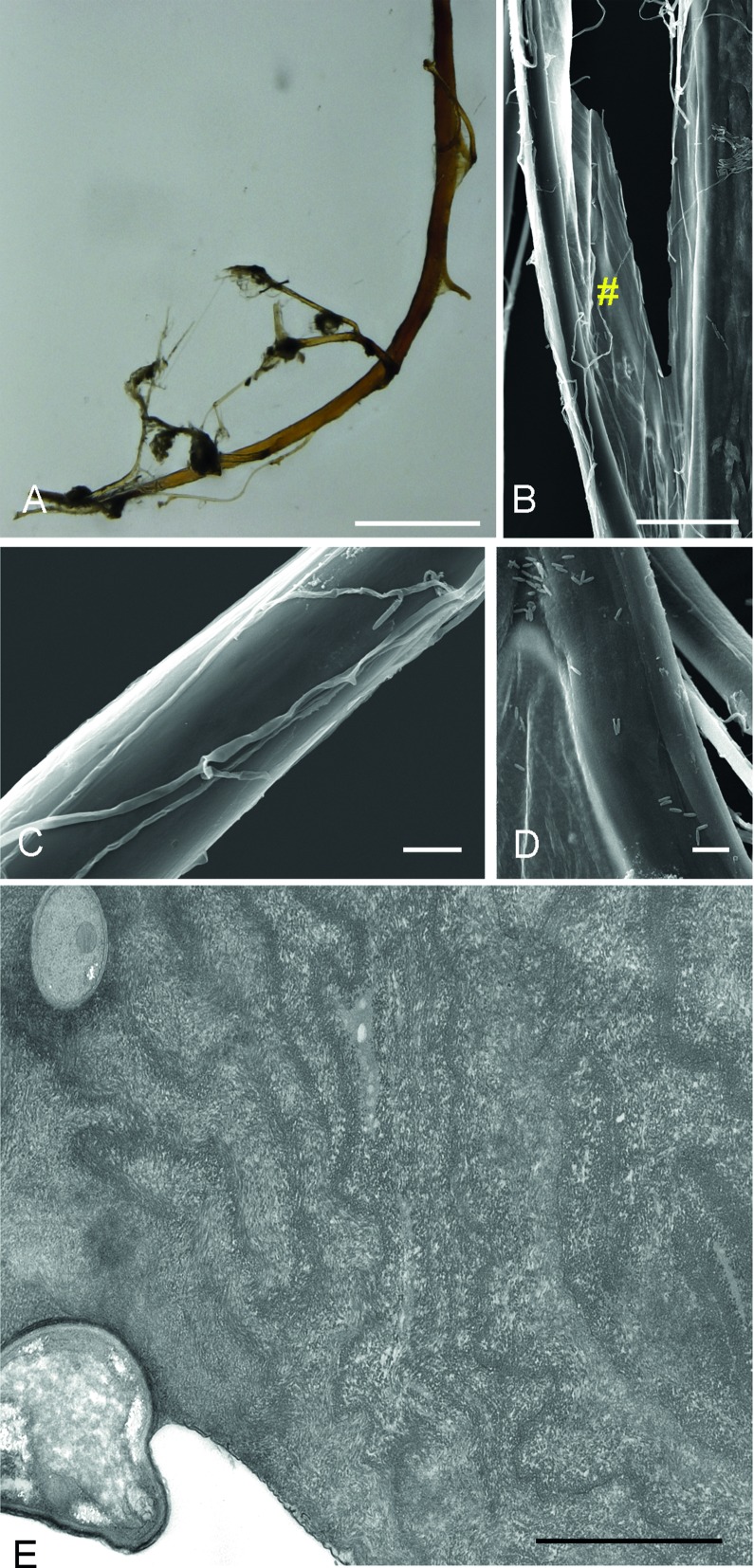
**A)** The suspensory cord of the *A*. *luminosa*, with which the pupa attaches to the substratum, has a root-like appearance. **B)** The strands are wrapped in a thin membrane (yellow hashtag) near the thorax base but separate with further progress. On several strands **C)** fungal fibres and **D)** rod-shaped bacteria could be observed. **E)** In the TEM, bacteria could also be observed within the cord and fungal fibres were attached to the cord. Scale bar in A = 1 mm; B = 50 μm; C and D = 10 μm; E = 2 μm.

The cord is used by the pupa to attach itself to the substratum during metamorphosis. It is dark in colour, stiff and relatively thick in diameter (≈ 100 μm) at its base near the pupa thorax and consists of several singular strands (Fig 11A) with a root-like appearance that the pupa attaches to the cave wall substratum. The cord lacks the droplets present on the fishing lines and feels dry when touched with a finger. The strands (diameter of ≈ 3–4 μm) are wrapped in a thin membrane at the base at the thorax (Fig 11B), but divide and form a plaque point (≈ 25–30 μm) near the anchor point at the substratum. Fungal fibres (Fig 11C) and rod-shaped bacteria (Fig 11D) were visible on several of the suspensory cord samples investigated, however, the pupa cuticle itself was not contaminated. In the TEM, bacteria could be observed in between the strands and there were fungi fibres attached to the cord (Fig 11E).

### Thread reproducibility

Behavioural observation indicates that a glowworm is able to rebuild a nest within a short period. One larva in Spellbound, whose nest was destroyed during thread isolation, crawled up the wall a few inches to another niche. Within twenty minutes it had rebuilt a new nest and produced two fishing-lines, each 3 cm long.

**Fig 12:**
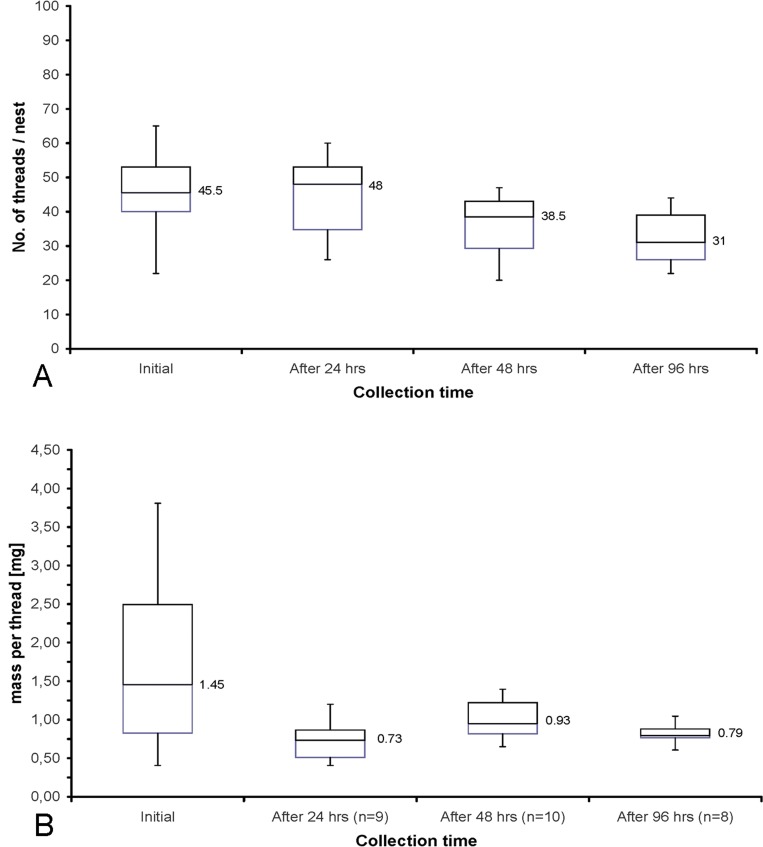
**A)** There is a complete reproduction of all threads in a nest within 24 h (from 45.5 threads initially to 48 threads). With continuous collection (48–96 h), the animals produce fewer threads. **B)** However, while the number of threads remains constant over 24 h, the weight of the individual threads decreases significantly, from 1.45 mg to 0.73 mg per thread. Also, with continuous collection (48–96 h), the weight of the reproduced threads remains low (0.93 mg or 0.79 mg) in relation to the weight of the initially collected thread.

Tests indicated that the larvae (n = 10) were not only able to reproduce the amount of threads in a nest completely within 24 h, but also increased the number of threads over time (increasing from 45 threads initially to 48 threads after 24 h (Fig 12A). With continuous collection, the number of threads reduced to 38.5 after 48 and then to 31 after 96 h. In contrast, while the number of threads increased within 24 h (Fig 12B), the weight of the threads decreased by 50% from 1.45 mg initially to 0.7 mg per thread after 24 h. However, with further thread collection, an increase to 0.93 mg after 48 h and finally to 0.79 mg after 96 h could be measured. In many cases the animals migrated away from the collection site because of this disturbance and a new animal and nest had to be chosen for the test series.

## Discussion

Although the impression of a star-spattered sky is surely the main reason why the renowned Waitomo caves on the North island of New Zealand have attracted so many tourists over the past 120 years [12; 59; 60; 61; 62], it is the fishing lines that attract the attention of visitors upon closer observation (pers. observation by the first and last author). Numerous biological and ecological studies have been performed in the past, explaining in detail the formation and usage of this prey-capture feature [16; 30; 31; 37; 63; 64; 65; 66; 67]. In this study we aim, in particular, to provide a close-up of their structure and an understanding of the droplet formation.

### Threads

The rapid replenishment of the removed fishing lines showed that larvae can tolerate a certain degree of web destruction, while continued disturbance causes the larvae to leave the area. It could therefore be assumed that unintended contact or removal of single threads by tourists or cave guides does not have a strong direct impact in the short term, as long as the nest remains intact. However, repeated removal should be avoided, as should other disturbance variables (light, noise, low humidity, etc) [67].

Earlier studies on thread composition have shown that the fishing lines in the glowworm *Arachnocampa* consist of cross-β sheet silk type [32; 33; 35], which occurs in a few other species (i.e. *Chrysopa carnea* [68; 69; 70]; *Hydrophilus* spp. [71] or *Hypera* spp. [72]), where it is used for different functions (egg stalking, reproduction and protection) and originates from different sites (colleterial gland) [32]. The silk fibers in *C*. *carnea* consist of one thread only and show intensive birefringence in polarized light [68; 69], whereas in *A*. *luminosa* it is presumed that several strands form the capture thread as observed in the SEM, which show no birefringence at all.

It remains questionable whether the pupa suspensory cord in *Arachnocampa* is likewise made of the same material as the fishing lines, in particular as the cord strands appeared narrower in diameter and showed no wettability or crystallisation in a dry state. In an earlier study it was proposed that the hammock is used as raw material for the suspensory cord [73], however a clear observation of the pupation process is so far lacking.

### Droplet origin

Gatenby (1959) assumed that the mucous droplets are formed in the paired cecae present in the oesophageal region and then regurgitated with the silk threads that are derived from the salivary glands. The enclosure of the crystals at the thread, observed in this study, may be a result of this regurgitation movement and enable a strong binding of the droplet core to the thread. The presence of diverse mineralised crystals in the droplets raises the question of whether the mucus is exclusively produced by specialized cells in the oesophagus region and whether it could incorporate parts of the primary urine. In most insects the primary urine produced in the Malphigian tubules shows a high abundance of ions such as Na^+^, K^+^, Cl^-^, Mg^2+^, KCL, SO_4_^2-^ and HCO_3_^-^ [74]. In *Arachnocampa*, just as in other insects, the primary urine is produced in the Malpighian tubules and passes into the hindgut to be removed in the faeces. It is possible that some urine components could be directed anteriorly and incorporated into the mucus, but this requires further investigation.

### Droplet formation

Earlier studies have shown that the caves and their local climate, in particular the temperature and humidity, have a large impact on the life cycle and mortality of glowworms [16; 38; 39; 65; 75]. Larvae are particularly vulnerable to desiccation because they secrete relatively large volumes of water into the mucus droplets arrayed on their webs. They re-ingest the mucus when removing old or tangled lines. Loss of moisture through evaporation under dry conditions could exceed their ability to replace it, leading to death by dehydration. The present data indicate that the saturation deficit could also influence the formation of droplets. The hygroscopic nature of the droplets could enable extraction of water from the environment into the droplets and thereby into the larval gut when lines are replaced. Based on the hygroscopic nature of the mineralized core and the fast process of dehydration and rehydration under artificial conditions, we assume that the hydrate layer formation around a droplet core takes place during the exposure of the threads in the highly humid caves and not earlier in the animal’s mouth. Long-term monitoring of both caves would be useful to verify humidity/temperature differences between them and to indicate a correlation with the thread weight and droplet amount. Moreover, the influence of tourists passing below the glowworm nests needs to be investigated in greater detail, concerning thread number and weight, and compared with those in a tourist-free area.

### Droplet composition

The drying experiments in the present study indicated that a droplet consists of 99% volatile substances, presumed to be primarily water, and around 1% of a hygroscopic and adhesive biomolecule, which becomes crystalline in a dry state (at a humidity < 80%). In the past it was assumed that glowworms use oxalic acid as a poison in their fishing lines to kill the prey [40], but later analysis by 37 could not detect the presence of this acid. In addition, the present EDX analyses speak against the presence of oxalic acid, as nitrogen was present in the crystal samples of all three *Arachnocampa* species and the carbon amount was lower in relation to the oxalic acid EDX spectra. The other two substances tested (urea and uric acid), which are commonly known to be waste products in insects [74; 76; 77; 78] showed congruence in terms of the carbon value to those of the crystal. However, in urea and uric acid, significantly higher nitrogen and oxygen values were given in relation to the data given for the crystals of *A*. *luminosa* and *A*. *tasmaniensis*. In *A*. *richardsae* a high nitrogen amount was observed, however the crystal contain in contrast a much lower oxygen values than the two tested substances.

Histochemical analyses failed to detect carbohydrates, lipids or proteins in the droplet remnants, however XPS data indicated the presence of peptides in the droplet, to which urea strongly binds.

Analyses of the Malphigian tubules composition in *A*. *luminosa* by Green (1979) resulted in histochemical reactions to calcium, magnesium, phosphates and urates (urea was not tested in this study) in laminated spherites within the cell type II tubule segment, while the other segment cell types (I; III and IV) remained unstained [15]. Based on the present data it is very likely that oxalic acid was absent in the droplet from *Arachnocampa*, however further investigations are necessary to determine whether urea and/or uric acid may have been present instead. Urea is commonly used in the bonding industry (mainly cardboard and wood manufacture) in combination with formaldehyde to form a non-transparent resin (UF resin), [79]. Therefore, it could not be excluded that the adhesive droplet in *Arachnocampa* also contained and urea-based glue and that, instead of formaldehyde, the yet-unknown peptide may have contributed as a cross-linker. So far, nothing is known about the usage of uric acid as an adhesive component in nature or industry. Therefore, further analyses are mandatory to fully understand the glue composition in glowworms to provide knowledge about its mechanisms.

### Comparison of the glowworm fishing lines with the capture threads of orb-spiders

As particularly the ecribellar prey capture system of orb-weaver spiders is in its outer appearance and functionally very close to the glowworm fishing lines, we provide a short comparison of the two systems: Like glowworms, orb spiders also use **silk threads** with regularly-spaced droplets for prey capture [51], however the spider’s silk type is organized in parallel-β [32]. Two different gland types are involved in creating this silk thread type, the flagelliform glands form the thread core of the capture spiral, while the adhesive coating is produced by the aggregate glands [80]. As mentioned earlier, the *Arachnocampa* fishing line is composed of a cross-β sheet silk type [32; 33; 35] and produced by paired labial glands [73]. *Argiope argentata* can produce seven different silk types with different mechanical properties and molecular structures [81]. It remains to be verified whether the glowworm is capable of producing different silk types for different purposes such as capture threads, anchorage lines and the pupal suspensory cord.

In both spiders and glowworms the **adhesive droplets** on the capture threads are regularly spaced; however, there are large differences in droplet distance, size and fluid dynamics: in spiders the droplets have a mean space of between 20 μm in *Leucauge venusta* to 264 μm in *Araneus marmoreus* [48]. The droplets are almost roundish, with a size (height x width) ranging from 4.4 x 4.5 μm in *Mangora maculata* to 67 x 50 μm in *A*. *marmoreus* [48; 49; 50]. Also, at low humidities (>20%) the droplets are still visible and adhesive in spiders [51; 82; 83; 84].

In *A*. *luminosa* the droplets are more ellipsoid-shaped, with dimensions of up to 1185 x 785 μm and at a distance of around 125 μm. Also, at a relative humidity of around 60% the droplets are hardly noticeable and show no bonding ability (unpublished data by the first author).

For the orb-weaver spiders a **droplet model** with three regions is proposed: “a small central, opaque anchoring granule (either elliptical or rectangular in shape, depending on the species [48]), a larger, surrounding, transparent glycoprotein glue region, and a more fluid outer covering that extends into the interdroplet regions” [51]. The glycoprotein core consists of *O*-linked N-acetylgalactosamine [51; 52; 85; 86], lipids [87] as well as organic and inorganic low-molecular-mass (LMM) compounds as Choline, GABamide, Isethionate and others [84; 88] but also inorganic salts as KNO_3_ [89], H_2_PO_4_^-^, Na^+^, Cl^-^ and Ca^2+^ [90]. However this coating is not only used to capture the prey, the LMM compounds absorb and hold up water from the air to keep the silk fibre extensible and the droplet in liquid form [90]. The orb-spider droplets however are likewise weakly acidic (pH 4) [89] as measured in the present study for the *A*. *luminosa* droplets.

In *A*. *luminosa* the droplets seem to have a two-layered organization, with a small core anchored to the capture thread and a thick outer water-based hull. In contrast to the orb-spiders, the core in *Arachnocampa* does not contain a clearly visible granule, but is only visible as amorphous crystals in a dried state. Based on the present data, a glyco- or lipoprotein core seems to be absent in the glowworm droplet, however further investigations regarding the peptides detected by XPS are planned.

## Summary

The present study indicates that the capture system in *Arachnocampa* strongly differs from that of orb spider webs regarding droplet organization, hygroscopic properties and core composition. This is not surprising given that orb spiders build their webs in exposed, desiccation-prone sites while *Arachnocampa* can only survive in sites with high humidity such as caves or wet forests. The differences are not only habitat-related, but probably also related to the origin of the web components; in *Arachnocampa* the fishing line silk is released from the labial glands into the mouth and the droplets are presumably released from the midgut. The urea and the mineralised crystals found in the droplets in this study could possibly be derived from excretory products.

While all of the *Arachnocampa* species have a common requirement for high-humidity environments, some species, notably *Arachnocampa luminosa* and *Arachnocampa tasmaniensis* seem to be more cave-adapted than others [91], reaching very high population numbers in caves and producing very long capture threads [16]. The differences in droplet composition seen among the three species investigated here (*Arachnocampa luminosa*, *Arachnocampa richardsae*, *Arachnocampa tasmaniensis*) could be related to their different core habitats and should be investigated further.
